# Norovirus GII.17 as Major Epidemic Strain in Italy, Winter 2015–16

**DOI:** 10.3201/eid2307.161255

**Published:** 2017-07

**Authors:** Giovanni Maurizio Giammanco, Simona De Grazia, Floriana Bonura, Vincenzo Cappa, Sara Li Muli, Arcangelo Pepe, Maria Cristina Medici, Fabio Tummolo, Adriana Calderaro, Francesca Di Bernardo, Piera Dones, Anna Morea, Daniela Loconsole, Cristiana Catella, Valentina Terio, Krisztiàn Bànyai, Maria Chironna, Vito Martella

**Affiliations:** Università di Palermo, Palermo, Italy (G.M. Giammanco, S. De Grazia, F. Bonura, V. Cappa, S. Li Muli, A. Pepe);; Università degli Studi di Parma, Parma, Italy (M.C. Medici, F. Tummolo, A. Calderaro);; ARNAS Ospedale Civico e Di Cristina, Palermo (F. Di Bernardo, P. Dones);; Università Aldo Moro di Bari, Bari, Italy (A. Morea, D. Loconsole, M. Chironna);; Università Aldo Moro di Bari, Valenzano, Italy (C. Catella, V. Terio, V. Martella);; Hungarian Academy of Sciences, Budapest, Hungary (K. Bànyai)

**Keywords:** Norovirus, gastroenteritis, viruses, enteric infections, Italy, GII.17 Kawasaki 2014

## Abstract

In winter 2015–16, norovirus GII.17 Kawasaki 2014 emerged as a cause of sporadic gastroenteritis in children in Italy. Median patient age was higher for those with GII.17 than GII.4 infection (55 vs. 24 months), suggesting limited cross-protection for older children.

Noroviruses are a major cause of acute gastroenteritis in children and adults. Although >30 norovirus genotypes can infect humans, the genotype GII.4 has been associated with most norovirus-related outbreaks and sporadic cases of gastroenteritis since the mid-1990s. Norovirus strains undergo genetic and antigenic evolution through accumulations of point mutations and intragenotype and intergenotype recombination.

During the winter season 2014–15, a novel NoV GII.P17_GII.17 variant, Kawasaki 2014, emerged in several countries in Asia, replacing the previously dominant variant, GII.4 Sydney 2012 (*1**–**4*). Kawasaki 2014 is phylogenetically distinct (Kawasaki-308 lineage) from earlier GII.17 variants that circulated in 2013 and 2014 and from the original 1978 GII.17 strain, which had a GII.4 open reading frame (ORF)1 gene (*4*). The spread of the variant Kawasaki 2014 in Asia was unexpected because this was the first time a variant other than GII.4 acquired such epidemiologic relevance. Until now, the Kawasaki 2014 variant had been reported in a limited number of cases in countries outside of Asia (*5*,*6*), including some in Europe (*6*–*8*), with most cases occurring sporadically.

The Italian Study Group for Enteric Viruses (http://isgev.net) monitors the epidemiology of enteric viruses in children through hospital-based surveillance in 3 geographically distinct areas: northern Italy, in Parma (University Hospital); southern Italy, in Bari (Pediatric University Hospital “Giovanni XXIII”); and on Sicily island, in Palermo (ARNAS Civic Hospital). The official total pediatric population (0–14 years of age) includes 426,569 newborns, infants, and children (as of January 1, 2015). 

From January 2015 through February 2016, the Italian Study Group for Enteric Viruses analyzed 2,603 fecal samples of children (0–14 years of age) with diarrhea. The collection of fecal samples was part of the process of diagnosis of acute gastroenteritis, and we obtained verbal informed consent for analysis from their families or caretakers. Norovirus prevalence was 12.8% (95/740) in Parma, 7.5% (95/1,273) in Bari, and 21.4% (126/590) in Palermo, yielding a calculated norovirus national prevalence of 12.1% (316/2,603). 

We performed multitarget analysis in the diagnostic regions A (ORF1, polymerase) and C (ORF2, capsid) of the norovirus genome (*9*). We characterized a subset (57.6%; 182/316) of the 2015–16 norovirus-positive samples either completely (44.0%; 139) or partially (13.6%; 43). Of the latter, 6 were characterized only in diagnostic region A (327 nt, at positions 4538–4865 relative to U07611 reference) and 37 only in diagnostic region C (342 nt, at positions 5307–5649 relative to U07611 reference) (Figure). 

In 2015, norovirus GII.P17_GII.17 Kawasaki 2014 was detected in 2 cases in February (*8*) and 1 in September, accounting for only 3.3% of the 90 norovirus positive samples from 2015 that were fully typed; the recombinant strain GII.P4 New Orleans 2009_GII.4 Sydney 2012 (38.9%; 35/90) and the pandemic variant GII.Pe_GII.4 Sydney 2012 (32.2%; 29/90) were predominant. Conversely, of the 49 strains fully typed for January and February 2016, the variant Kawasaki reached 18.4% (9/49) prevalence and represented the third most common strain in Italy, after GII.P4 New Orleans 2009_GII.4 Sydney 2012 (34.7%; 17/49) and GII.Pe_GII.4 Sydney 2012 (24.5%; 12/49). Occurrence of related cases or outbreaks was ruled out on the basis of the patients’ anamnestic data. 

We determined the sequence of a large portion of the genome at the 3′ end (3.2-kb, including partial ORF1 [822 nt], full-length ORF2 [1621 nt], and ORF3 [866 nt]) for 3 representative GII.P17_GII.17 strains from this study (GenBank accession nos. BA202/16–16: KX592170; PA31/2016: KX592171; and PA39/2016: KX592172). After phylogenetic analysis, the Kawasaki 2014 variants from Italy segregated into the Kawasaki-308 genetic subclade, together with other GII.P17_GII.17 sequences available in GenBank, including those of noroviruses detected in China and Hong Kong during 2014–15 and in the United States in November 2014 (*8*) (Technical Appendix).

Analysis of demographic data revealed a significantly higher median age for patients with GII.P17_GII.17 infections (55 [SD 49.8] months) than for patients with GII.4 infections (24 [SD 13.6] months) (p<0.005; 2-tailed Mann U-test, p = 0.00433 [95% CI 0.4–6.5]). These observations are consistent with a lack of specific herd immunity in the population, meaning that the GII.17 virus can infect older patients more easily than GII.4 viruses can, as observed in Hong Kong (*4*).

Our analysis indicates that, in Italy in winter 2015–16, the epidemiologic pattern of norovirus GII.17 viruses markedly changed, suggesting increased circulation of the variant Kawasaki 2014 among children, although GII.4 variants (the capsid variant Sydney 2012 with the GII.Pe or GII.P4 polymerase) were still predominant. The mechanisms driving the global spread of norovirus GII.17 could include the broad range of co-receptors used by these viruses (*10*) or the limited cross-antigenic relationships with the predominant GII.4 strains that could trigger mechanisms of antigenic escape. Norovirus GII.17 could present a challenge for the development of norovirus vaccines because it is not clear whether, and to what extent, there is cross-protection between vaccine antigens and GII.17 viruses (*6*).

**Figure:**
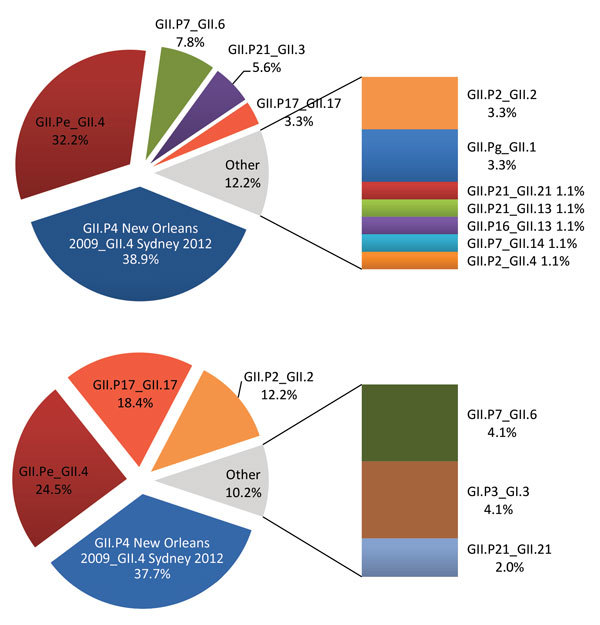
Norovirus genotypes for fully typed strains detected during January–December 2015 (A) and during January–February 2016 (B) in Italy by the Italian Study Group for Enteric Viruses surveillance system.

Technical Appendix Phylogenetic analysis of norovirus isolates from Italy, winter 2015–16.
