# Supplementary material for: Norovirus GII.17 as Major Epidemic Strain in Italy, Winter 2015–16
Source: Emerg Infect Dis. 2017 Jul;23(7):1206–8. doi: 10.3201/eid2307.161255 (PMC5512478; doi:10.3201/eid2307.161255)
Supplement: Technical Appendix — Phylogenetic analysis of norovirus isolates from Italy, winter 2015–16. [file 16-1255-Techapp-s1.pdf]

Article DOI: http://dx.doi.org/10.3201/eid2307.161255

Norovirus GII.17 as Major Epidemic Strain in Italy, Winter 2015–16

Technical Appendix

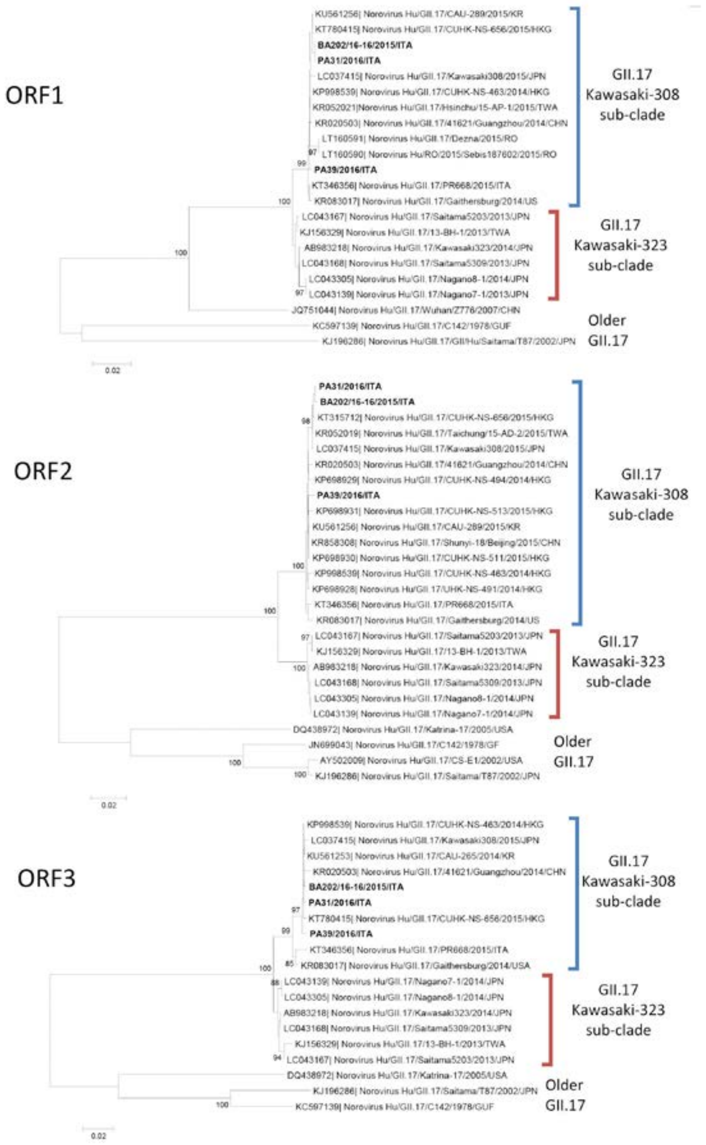

Technical Appendix Figure. Phylogenetic analysis based on partial sequence of ORF1 (822 nt), the full-length sequence of ORF2 (1621 nt) and ORF3 (866 nt) sequences of GII.P17_GII.17 noroviruses. The GII.P17_GII.17 strains from Italy are indicated in bold. The trees were built with the Neighbor-joining method and Kimura 2-parameter model, with bootstrapping over 1000 repetitions. Bootstrap values >80% are indicated. The scale bar indicates the number of nucleotide substitutions per site.
